# Exposure to second-hand smoke during early life and subsequent sleep problems in children: a population-based cross-sectional study

**DOI:** 10.1186/s12940-021-00793-0

**Published:** 2021-12-18

**Authors:** Li-Zi Lin, Shu-Li Xu, Qi-Zhen Wu, Yang Zhou, Hui-Min Ma, Duo-Hong Chen, Peng-Xin Dong, Shi-Min Xiong, Xu-Bo Shen, Pei-En Zhou, Ru-Qing Liu, Gongbo Chen, Hong-Yao Yu, Bo-Yi Yang, Xiao-Wen Zeng, Li-Wen Hu, Yuan-Zhong Zhou, Guang-Hui Dong

**Affiliations:** 1 Guangdong Provincial Engineering Technology Research Center of Environmental Pollution and Health Risk Assessment, Department of Occupational and Environmental Health, School of Public Health, Sun Yat-sen University, 74 Zhongshan 2nd Road, Yuexiu District, Guangzhou, 510080 China; 2 State Environmental Protection Key Laboratory of Environmental Pollution Health Risk Assessment, South China Institute of Environmental Sciences, Ministry of Environmental Protection, Guangzhou, 510655 China; 3 State Key Laboratory of Organic Geochemistry and Guangdong Key Laboratory of Environmental Protection and Resources Utilization, Guangzhou Institute of Geochemistry, Chinese Academy of Sciences, Guangzhou, 510640 China; 4 Department of Air Quality Forecasting and Early Warning, Guangdong Environmental Monitoring Center, State Environmental Protection Key Laboratory of Regional Air Quality Monitoring, Guangdong Environmental Protection Key Laboratory of Atmospheric Secondary Pollution, Guangzhou, 510308 China; 5 Nursing College, Guangxi Medical University, Nanning, 530021 China; 6 School of Public Health, Zunyi Medical University, 6 Xuefu Road, Xinpuxin District, Zunyi, 563060 China

**Keywords:** Secondhand smoke, Sleep, Children, Public health

## Abstract

**Background:**

Previous studies have revealed that current secondhand smoke exposure showed highly suggestive evidence for increased risk of simultaneous sleep problems in children. Data on the associations between early-life exposure to SHS with subsequent sleep problems in children were scarce. We aimed to evaluate the associations of early-life SHS exposure with sleep problems in children.

**Methods:**

In this cross-sectional study, children were recruited from elementary and middle schools in Liaoning Province, China between April 2012 and January 2013. We assessed early-life SHS exposure (pregnancy and the first 2 years of life) via questionnaires. Sleep problems and different types of sleep-related symptoms were measured based on the validated tool of the Sleep Disturbance Scale for Children (SDSC). Generalized linear mixed models were applied to estimate the associations of early-life SHS exposure with sleep problems.

**Results:**

We included a total of 45,562 children (22,657 [49.7%] males; mean [SD] age, 11.0 [2.6] years) and 6167 of them (13.5%) were exposed to early-life SHS during both pregnancy and the first 2 years of life. Compared with unexposed counterparts, children exposed to early-life SHS had higher total T-scores of SDSC (β = 4.32; 95%CI: 4.06, 4.58) and higher odds of increased sleep problems (OR = 2.14; 95%CI: 1.89, 2.42). When considering different sleep-related symptoms, the associations between early-life SHS exposure and symptom of sleep-wake transition disorders (i.e., bruxism) were the strongest in all analyses.

**Conclusions:**

Early-life SHS exposure was associated with higher odds of global sleep problems and different sleep-related symptoms in children aged 6–18 years. Our findings highlight the importance to strengthen efforts to support the critical importance of maintaining a smoke-free environment especially in early life.

**Supplementary Information:**

The online version contains supplementary material available at 10.1186/s12940-021-00793-0.

## Background

In 2019, China still accounted for more than one-third of the global tobacco use according to the latest Global Burden of Disease Study [1]. There are over 341 million smokers in China and the prevalence of smoking in Chinese men has reached 49.7% [1], indicating one important issue of exposure to second-hand smoke (SHS) in Chinese children [2]. Many studies, including ours, have already identified that SHS exposure during early-life caused numerous health consequences in children, including severe asthma attacks, impaired lung function and respiratory symptoms, neurodevelopmental disorders and so on [3–5]. Recent studies have linked SHS exposure to sleep problems in children, which has emerged as another public health issue due to the increasing prevalence worldwide [6]. It has been estimated that the pooled prevalence of sleep problems among children in mainland China was 37.6% and the prevalence of specific sleep-related symptoms varied greatly [7]. Since good sleep quality is a well-recognized predictor of physical and mental health during childhood and adolescence [8], it’s of importance to understand the associations between SHS exposure and sleep problems in children especially in China.

Most of the previous observational studies across different countries have evaluated current SHS exposure and simultaneous sleep problems in children (eTable.1–2 in the Supplement). Specifically, these studies focused on the associations of current SHS exposure with several sleep-related symptoms including initiating and maintaining sleep, sleep-breathing, day time sleepiness and night awakenings. However, the associations might be different when considering other types of sleep-related symptoms (parasomnias, sleep hyperhidrosis, etc.). Since they were seldom discussed in previous studies, detailed investigations of different types of sleep symptoms are still needed. Moreover, there has been minimal attention to the potential impact of SHS exposure in the early life (i.e., during pregnancy and the first 2 years of life), and we only found two cohort studies (the UK [9] and the USA [10]) addressing prenatal SHS exposure and symptom of sleep-breathing in children throughout early childhood. Mechanically, sleep behaviors are regulated by the central nervous system, therefore being sensitive to alterations in brain neurochemistry [11]. Chemicals in SHS can cross placental barrier during pregnancy and disrupt neurochemistry or influence the development of infants’ brain structure by interfering with the breathing process [11]. However, to our knowledge, no studies include measures of early-life SHS exposure during both pregnancy and the first 2 years of life.

Therefore, the objective of the present study was to investigate the associations between early-life exposure to SHS and sleep problems in Chinese children. We improved on previous studies by using a more comprehensive and validated measurement of sleep problems, and parent-reported SHS exposure during early life. We hypothesized that the associations between SHS exposure and sleep problems might be different when considering different types of sleep-related symptoms and the exposure timing of early-life.

## Methods

### Study population and overall design

This cross-sectional study was embedded in the second wave of the Seven Northeastern Cities study between April 2012 and January 2013. The sampling strategy was developed as follows: a representative sample of Liaoning province located in Northeastern China was generated by randomly selecting half of the 14 cities in this province. One elementary school and one middle school was randomly chosen in 24 urban districts from the selected seven cities. From each grade level of the included schools, we invited students of one or two classrooms to participate in this study, who lived in the study area for at least 2 years before the start of this study. Finally, a total of 48,612 eligible children and adolescents aged 6 to 18 years have participated in this survey, and 45,562 of them have completed the assessments of sleep behaviors. This study was approved by the Ethical Review Committee for Biomedical Research, Sun Yat-sen University. We declared that we followed the Strengthening the Reporting of Observational Studies in Epidemiology (STROBE) reporting guideline for cross-sectional studies.

### Procedures

In each school, we organized face-to-face appointments for the teachers and the principals to introduce the aims, proposed methods and the procedures of our study. We aimed to incentivize permission for parents by using active communication techniques with the help of the teachers and the principals. We provided standard procedures for the school teachers. School teachers were required to explain the study aim, obtain the informed consent, and distribute the questionnaires in regular parent-teacher conferences. Parents could fill out the questionnaires during the conference or take it home and return it in a sealed envelope. Parents had the right to decline to consent and refuse to join the study.

### Parent-reported sleep disturbance and related problems of their children

We asked parents to fill out the Sleep Disturbance Scale for Children (SDSC) to measure sleep quality in all children. The SDSC was a 26-item parent-rated scale developed by Oliviero Bruni in 1996 [12]. Each item was rated on a 5-point Likert scale: 1 = never; 2 = occasionally (once or twice a month); 3 = sometimes (once or twice a week); 4 = often (three to five times a week) and 5 = always (six or seven times a week). The SDSC provides a total score of sleep disturbance and six domain scores including: (1) disorders of initiating and maintaining sleep (DIMS), such as sleep duration, sleep latency, night awakenings, and anxiety falling asleep; (2) sleep breathing disorders (SBD), such as snoring and breathing problems; (3) disorders of arousal (DA), such as sleepwalking, sleep terrors, and nightmares; (4) sleep–wake transition disorders (SWTD), such as rhythmic movements, hypnic jerks, sleep talking, and bruxism; (5) disorders of excessive somnolence (DOES), such as difficulty waking up, morning tiredness, and inappropriate napping; and (6) sleep hyperhidrosis (SHY), such as nocturnal sweating. The total score and the subscale scores can then be converted to a T-score so that we can compare them among children in different age groups. We also used validated cut-offs to yield proxies for sleep disturbance and increased problems in the six domains within the clinical range (i.e., T-scores ≥70). In addition, both sleep duration and sleep latency could be derived based on two of the items in the SDSC. We defined inappropriate sleep duration (i.e., < 7 h) and sleep latency (i.e., > 45 min) according to the international consensus recommendations [13, 14].

We have already conducted a validation study to revise the Chinese version of SDSC in the first wave of the Seven Northeastern Cities study, and it is reliable in screening parent-reported sleep problems in Chinese children (Cronbach’s α = 0.81). The detail of the study is described elsewhere [15].

### Assessment of SHS exposure

We collected information on SHS exposure via questionnaires. We defined having exposure to early-life SHS based on an affirmative answer to the two questions: (1) Did anyone who lived with the mother during her pregnancy smoke anywhere inside the house? and (2) Did anyone who lived with the child during his or her first 2 years smoke anywhere inside the house? Therefore, exposure to early-life SHS was a category variable encoded as (1) unexposed; (2) ever exposed during pregnancy or the first 2 years of life; and (3) ever exposed during both pregnancy and the first 2 years of life.

We also collected information on the current number of cigarettes smoked inside the house per day during weekdays and weekends by all family members who lived with the child, and therefore we defined having current SHS exposure if any family member who lived with the child smoked cigarettes.

### Statistical analyses

We conducted data analyses from April 32,021 to May 3, 2021. We calculated means and standard deviations for continuous variables and percentages for categorical variables. The differences across different SHS exposure groups were determined using ANOVA test for continuous variables and chi-square tests for categorical variables.

We analyzed the associations of exposure to early-life SHS with sleep problems in children by fitting generalized linear mixed models with an identity link (continuous outcomes with gaussian distribution) or logit link (binary outcomes with binomial distribution) function. We fitted crude models with school as random intercept. We fitted adjusted models for each outcome, adjusting for covariates collected from questionnaires (child age, sex, only child, preterm birth and low birth weight, parental educational levels, yearly household income, maternal age during pregnancy, maternal smoking and alcohol consumption during pregnancy). We conducted stratified analyses by sex and by child age, and whether the associations varied with sex or child age were assessed from the heterogeneity of effect across strata and the significance of interaction terms.

To verify the robustness of the results, sensitivity analyses were performed: (1) we examined the associations by excluding children with parent-reported asthma (*n* = 2257, 4.95%) since previous studies have found strong associations between SHS exposure and sleep problems in asthmatic children [16]; (2) we grouped SHS exposure into more detailed categories of exposure by considering pregnancy and the first 2 years of life separately and re-analyzed the data; (3) we used current SHS exposure derived from the questionnaires to confirm the associations between current SHS exposure and simultaneous sleep problems since most of previous studies have confirmed the above association.

Statistical analyses were conducted with the statistical software R 4.0.3 (R Core Team 2020). We presented the results as estimates (β) and odds ratios (OR) with the 95% confidence interval (CI). A *P* value < 0.05 for two-sided test was considered statistically significant.

## Results

### Characteristics of the study population

As shown in Table 1, 34,642 of the 45,562 children (76.0%) were not exposed to SHS during early life; 4753 of them (10.4%) were ever exposed during pregnancy or the first 2 years of life; and 6167 of them (13.5%) were ever exposed during both pregnancy and the first 2 years of life. There were significant differences among children in different SHS groups in all characteristics. The total score of sleep problems measured by SDSC was 40.0 ± 8.6 with no sex difference, and the prevalence of short sleep duration and long sleep latency were 7.0 and 1.2%, respectively. The detail for the six domain scores was shown in eTable.3.

**Table 1:** General characteristics of 45,562 study participants^a^

Characteristics	Overall	Exposure to SHS during early life			*P* value
		Unexposed (*n* = 34,642)	Ever exposed during pregnancy or the first 2 years of life (*n* = 4753)	Ever exposed during both pregnancy and the first 2 years of life (*n* = 6167)	
Child age, years	11.0 (2.6)	11.0 (2.6)	11.2 (2.6)	11.4 (2.5)	**< 0.01**
Child sex					
Male	22,657 (49.7)	17,310 (50.0)	2382 (50.1)	2965 (48.1)	**0.02**
Female	22,905 (50.3)	17,332 (50.0)	2371 (49.9)	3202 (51.9)	
Only child					
Yes	39,212 (86.1)	29,934 (86.4)	4067 (85.6)	5211 (84.5)	**< 0.01**
No	6350 (13.9)	4708 (13.6)	686 (14.4)	956 (15.5)	
Preterm birth					
Yes	2349 (5.2)	1660 (4.8)	300 (6.3)	389 (6.3)	**< 0.01**
No	43,213 (94.8)	32,982 (95.2)	4453 (93.7)	5778 (93.7)	
Low birth weight					
Yes	1777 (3.9)	1300 (3.8)	215 (4.5)	262 (4.2)	**< 0.01**
No	43,785 (96.1)	33,342 (96.2)	4538 (95.5)	5905 (95.8)	
Parental educational levels					
Low	29,113 (63.9)	21,146 (61.0)	3334 (70.1)	4633 (75.1)	**< 0.01**
High	16,449 (36.1)	13,496 (39.0)	1419 (29.9)	1534 (24.9)	
Yearly household income					
≤ 10,000 Yuan	10,117 (22.2)	7393 (21.3)	1094 (23.0)	1630 (26.4)	**< 0.01**
10,001–30,000 Yuan	17,817 (39.1)	13,217 (38.2)	1970 (41.4)	2630 (42.6)	
30,001–10,000 Yuan	15,346 (33.7)	12,156 (35.1)	1488 (31.3)	1702 (27.6)	
> 100,000 Yuan	2282 (5.0)	1876 (5.4)	201 (4.2)	205 (3.3)	
Maternal age					
≤ 25 years old	21,093 (46.3)	15,598 (45.0)	2391 (50.3)	3104 (50.3)	**< 0.01**
25–30 years old	20,376 (44.7)	16,063 (46.4)	1893 (39.8)	2420 (39.2)	
30–35 years old	3002 (6.6)	2221 (6.4)	333 (7.0)	448 (7.3)	
> 35 years old	1091 (2.4)	760 (2.2)	136 (2.9)	195 (3.2)	
Maternal smoking during pregnancy					
Yes	286 (0.6)	52 (0.2)	66 (1.4)	168 (2.7)	**< 0.01**
No	45,276 (99.4)	34,590 (99.8)	4687 (98.6)	5999 (97.3)	
Maternal alcohol consumption during pregnancy					
Yes	340 (0.7)	133 (0.4)	64 (1.3)	143 (2.3)	**< 0.01**
No	45,222 (99.3)	34,509 (99.6)	4689 (98.7)	6024 (97.7)	

*Abbreviation*: *SHS* Second-hand smoke

^a^Data are mean (standard deviation) for continuous variables and number (percentage) for categorical variables

### Associations between early-life SHS exposure and sleep problems

In the adjusted model (Table 2), compared with unexposed counterparts, children ever exposed during pregnancy or the first 2 years of life had higher total T-scores of SDSC (β = 2.69; 95%CI: 2.40, 2.98), and children ever exposed during both pregnancy and the first 2 years of life had the highest total T-scores of SDSC (β = 4.32; 95%CI: 4.06, 4.58). Similarly, children with early-life SHS exposure had higher T-scores in the six domains of the SDSC with the estimates ranging from 1.24 to 4.09, and we observed the strongest associations in the analyses of SWTD T-score (β = 4.09; 95%CI: 3.82, 4.35 for children ever exposed during both pregnancy and the first 2 years of life).

**Table 2:** Associations of SHS exposure during early life with the scores of the SDSC in children aged 6–18 years^a^

Estimates (95%CI)	Exposure to SHS during early life		
	Unexposed	Ever exposed during pregnancy or the first 2 years of life	Ever exposed during both pregnancy and the first 2 years of life
Total score			
Model 1	[1] Reference	2.84 (2.55, 3.13)	4.56 (4.30, 4.82)
Model 2	[1] Reference	2.69 (2.40, 2.98)	4.32 (4.06, 4.58)
DIMS score			
Model 1	[1] Reference	1.72 (1.43, 2.00)	3.05 (2.80, 3.31)
Model 2	[1] Reference	1.56 (1.27, 1.84)	2.81 (2.55, 3.07)
SBD score			
Model 1	[1] Reference	1.38 (1.09, 1.67)	2.33 (2.07, 2.59)
Model 2	[1] Reference	1.34 (1.05, 1.63)	2.30 (2.03, 2.56)
DA score			
Model 1	[1] Reference	1.48 (1.19, 1.78)	2.19 (1.92, 2.45)
Model 2	[1] Reference	1.34 (1.05, 1.63)	1.96 (1.70, 2.23)
SWTD score			
Model 1	[1] Reference	2.76 (2.47, 3.06)	4.23 (3.96, 4.49)
Model 2	[1] Reference	2.68 (2.38, 2.97)	4.09 (3.82, 4.35)
DOES score			
Model 1	[1] Reference	2.21 (1.91, 2.51)	3.42 (3.16, 3.69)
Model 2	[1] Reference	2.07 (1.77, 2.37)	3.20 (2.93, 3.47)
SHY score			
Model 1	[1] Reference	1.22 (0.94, 1.50)	2.07 (1.82, 2.32)
Model 2	[1] Reference	1.24 (0.96, 1.52)	2.12 (1.87, 2.37)

*Abbreviations*: *CI* Confidence interval, *DA* Disorders of arousal, *DIMS* Disorders of initiating and maintaining sleep, *DOES* Disorders of excessive somnolence, *SBD* Sleep breathing disorders, *SDSC* The Sleep Disturbance Scale for Children, *SHS* Second-hand smoke, *SHY* Sleep hyperhidrosis, *SWTD* Sleep–wake transition disorders

^a^Model 1 was adjusted for a school-level random intercept. Model 2 was adjusted for a school-level random intercept, child age, sex, only child, preterm birth and low birth weight, parental educational levels, yearly household income, maternal age during pregnancy, maternal smoking and alcohol consumption during pregnancy

When using the cut-offs to analyze the associations between early-life SHS exposure and the proxies for increased problems in the total scores of SDSC and six domains within the clinical range, we found similar results compared with those using the continuous T-scores (Table 3). For example, compared with unexposed counterparts, children ever exposed during both pregnancy and the first 2 years of life had higher odds of increased sleep problems (OR = 2.14; 95%CI: 1.89, 2.42). We also observed the strongest associations in the analyses of increased SWTD (OR = 1.97; 95%CI: 1.76, 2.20 for children ever exposed during both pregnancy and the first 2 years of life). Meanwhile, when extracting the information of short sleep duration and long sleep latency, we only found that early-life SHS exposure was associated with higher odds of long sleep latency (e.g., OR = 1.54; 95%CI: 1.24, 1.92 for children ever exposed during both pregnancy and the first 2 years of life).

**Table 3:** Associations of SHS exposure during early life with the increased problems within the clinical range using the SDSC in children aged 6–18 years^a^

Odd Ratios (95%CI)	Exposure to SHS during early life		
	Unexposed	Ever exposed during pregnancy or the first 2 years of life	Ever exposed during both pregnancy and the first 2 years of life
Increased sleep problems			
Model 1	[1] Reference	1.91 (1.65, 2.21)	2.43 (2.15, 2.74)
Model 2	[1] Reference	1.76 (1.52, 2.04)	2.14 (1.89, 2.42)
Increased DIMS			
Model 1	[1] Reference	1.52 (1.30, 1.78)	1.99 (1.75, 2.27)
Model 2	[1] Reference	1.43 (1.22, 1.67)	1.78 (1.56, 2.04)
Increased SBD			
Model 1	[1] Reference	1.50 (1.33, 1.70)	1.80 (1.62, 2.00)
Model 2	[1] Reference	1.48 (1.31, 1.67)	1.77 (1.59, 1.97)
Increased DA			
Model 1	[1] Reference	1.53 (1.31, 1.78)	1.68 (1.47, 1.92)
Model 2	[1] Reference	1.42 (1.21, 1.66)	1.48 (1.29, 1.70)
Increased SWTD			
Model 1	[1] Reference	1.61 (1.41, 1.83)	2.13 (1.91, 2.37)
Model 2	[1] Reference	1.53 (1.34, 1.75)	1.97 (1.76, 2.20)
Increased DOES			
Model 1	[1] Reference	1.56 (1.39, 1.76)	1.99 (1.80, 2.20)
Model 2	[1] Reference	1.49 (1.32, 1.68)	1.85 (1.67, 2.05)
Increased SHY			
Model 1	[1] Reference	1.41 (1.24, 1.59)	1.73 (1.55, 1.92)
Model 2	[1] Reference	1.42 (1.25, 1.61)	1.77 (1.58, 1.97)
Shorter sleep duration			
Model 1	[1] Reference	1.08 (0.96, 1.22)	1.07 (0.96, 1.19)
Model 2	[1] Reference	1.06 (0.93, 1.20)	1.05 (0.94, 1.18)
Longer sleep latency			
Model 1	[1] Reference	1.65 (1.30, 2.10)	1.75 (1.41, 2.16)
Model 2	[1] Reference	1.53 (1.20, 1.96)	1.54 (1.24, 1.92)

*Abbreviations*: *CI* Confidence interval, *DA* Disorders of arousal, *DIMS* Disorders of initiating and maintaining sleep, *DOES* Disorders of excessive somnolence, *SBD* Sleep breathing disorders, *SDSC* The Sleep Disturbance Scale for Children, *SHS* Second-hand smoke, *SHY* Sleep hyperhidrosis, *SWTD* Sleep–wake transition disorders

^a^Model was adjusted for a school-level random intercept, child age, sex, only child, preterm birth and low birth weight, parental educational levels, yearly household income, maternal age during pregnancy, maternal smoking and alcohol consumption during pregnancy

The results were similar in stratified analyses by sex or by child age, and we only found significant modification of sex or child age on the associations of early-life SHS exposure with the T-score of DOES and/or SHY (Table 4). The association between SHS exposure and T-score of DOES was stronger in females (*P*_*interaction*_ = 0.0298), while the association between SHS exposure and T-score of SHY was stronger in males (*P*_*interaction*_ = 0.0253). When considering child age, the association between SHS and T-score of DOES was stronger in older children (*P*_*interaction*_ < 0.001).

**Table 4:** Associations of SHS exposure during early life with the T-scores of DOES and SHY stratified by sex or by child age^a^

	Exposure to SHS during early life			*P*_interaction_
	Unexposed	Ever exposed during pregnancy or the first 2 years of life	Ever exposed during both pregnancy and the first 2 years of life	
**Stratified by sex**				
DOES t-score				0.0298
Males	[1] Reference	1.82 (1.40, 2.24)	2.89 (2.50, 3.27)	
Females	[1] Reference	2.32 (1.90, 2.74)	3.50 (3.13, 3.88)	
SHY t-score				0.0253
Males	[1] Reference	1.58 (1.18, 1.97)	2.33 (1.97, 2.69)	
Females	[1] Reference	0.91 (0.51, 1.30)	1.92 (1.57, 2.26)	
**Stratified by child age**				
DOES t-score				< 0.001
6–11 years (primary school)	[1] Reference	1.61 (1.19, 2.04)	2.97 (2.58, 3.36)	
12–14 years (middle school)	[1] Reference	2.49 (2.04, 2.95)	4.69 (3.44, 5.94)	
15–18 years (high school)	[1] Reference	1.98 (1.32, 2.65)	4.65 (3.68, 5.63)	

*Abbreviations*: *CI* Confidence interval, *DOES* Disorders of excessive somnolence, *SHS* Second-hand smoke, *SHY* Sleep hyperhidrosis

^a^Model was adjusted for a school-level random intercept, child age, sex, only child, preterm birth and low birth weight, parental educational levels, yearly household income, maternal age during pregnancy, maternal smoking and alcohol consumption during pregnancy and interaction term between SHS exposure and sex/child age

### Sensitivity analyses

When grouping the early-life SHS exposure into two detailed categories (pregnancy and the first 2 years of life), the results remained similar (eTables.4 and 5). We repeated the analyzes by excluding asthmatic children and the effect estimates were similar to those in the main analysis (eTable.6). We also repeated the analyses using current SHS exposure, and the associations remained similar with smaller effect estimates effect (eTable.7).

## Discussion

In the present study, we found that early-life SHS exposure was associated with global sleep problems in children aged 6–18 years. Specifically, we found that the associations were stronger in children with symptoms related to SWTD. In addition, we found significant sex and age differences in the associations of early-life SHS exposure with the DOES and SHY T-score.

Most of the previous studies focused on studying the associations between current SHS exposure and one specific sleep-related symptom without considering the global sleep problems. We only found one cross-sectional study was conducted in Taiwan, China using a comprehensive measure of Pittsburg Sleep Quality Index (PSQI), and indicated a positive but null association between current SHS exposure and poor sleep quality in 213 adolescents (10–15 years) [17]. In our study, we have validated the Chinese version of SDSC to measure global sleep problems, and we confirmed that the associations were stronger in early life compared with those in current period, highlighting vulnerable exposure windows that can occur as early as the prenatal and early postnatal periods. Mechanistically, nicotine or cotinine (a major metabolite of nicotine) in tobacco smoke can easily cross the placental barrier when exposed prenatally [18]. Animal studies of neonatal rats provide experimental evidence that prenatal nicotine exposure impaired the central chemoreception of the central nervous system that control respiratory activity [19]. Postnatally, as a stimulant, nicotine can indirectly inhibit sleep-promoting neurons in the ventrolateral preoptic area of young rats via nicotinic presynaptic enhancement of noradrenaline release [20]. Meanwhile, the critical exposure window for the harmful effects of nicotine on fetal lung development is hypothesized to occur in the second and third trimester [21], and both prenatal and postnatal nicotine can impair upper airway neuromuscular protective reflexes in animal models [22]. Consistent with these mechanisms, our findings indicated that children’s sleep quality might benefit from effective and continuous interventions for smoking cessation targeting general population, which are still needed to protect children from SHS exposure especially during their early life.

Previous observational studies focused on studying sleep-related symptoms of DIMS, SBD, DA and DOES with inconsistent results. We have extended the findings by considering two more symptoms of SWTD and SHY, which were prevalent but seldom discussed in children. One randomized controlled study of 498 Italian children aged 8–11 years suggested that interventions on parental smoking reduced the prevalence of sleep bruxism (one symptom of SWTD) [23]. One community-based two-generation survey in Northern Europe, Spain and Australia provided first evidence that adult offspring population whose parents were ex-smokers had higher odds of nocturnal sweating (one symptom of SHY) [24]. Since relevant studies were scare, more studies are needed to understand the underlying mechanisms of above associations in children. Surprisingly, we were the first to observe the strongest associations between SHS exposure and children’s SWTD symptoms in all analyses when compared with others. Sleep bruxism is a rhythmic or non-rhythmic SWTD symptom that involves masticatory muscle activity during sleep, which is regulated by the central nervous system related to tooth contact [25]. The prevalence of sleep bruxism varied and decreased with age in children (3.5–40.6%), which were either ignored or unnoticed by the parents [26]. Therefore, we proposed that SWTD symptom especially sleep bruxism should be well considered in future studies.

In this study, we have identified significant sex differences in the associations between early-life SHS exposure and the T-score of DOES and SHY, but the potential mechanism remained unclear. Our results might be supported by previous animal data that lung function alterations in mice were sex-specific with males being more susceptible to early-life SHS exposure [27]. Moreover, steroid hormones might modulate sleep behaviors, and females typically report poorer sleep quality and more sleep disturbance across different stages of life [28]. For example, a cross-sectional study of 9261 school-aged Japanese children suggested that a higher proportion of females reported daytime sleepiness (a symptom of DOES) in comparison to males [29]. Specifically, when considering child age, we also found stronger SHS-DOES association in children with older age, especially for children in middle school. The pubertal change might delayed sleep phase and disrupted sleep patterns in middle school students [30], and stress of school performance might also contribute to poorer sleep for older adolescents [31]. Therefore, symptom of DOES might be more sensitive to early-life SHS in females and in older adolescents. Although few studies have investigated symptom of SHY, previous study also indicated that androgen deprivation therapy for prostate cancer is highly associated with an increased occurrence of night sweating [28]. However, interpretation should be cautious because the sex-specific associations might be resulted from the sex-specific response to nicotine exposure or the sex differences of sleep behaviors or both. Besides, we did not find sex-specific association between early-life exposure and the binary outcomes. Therefore, more studies are needed to understand the role of sex and child age on the associations between early-life SHS exposure and sleep problems.

### Strength and limitations

Several limitations should be well noted. First, due to the cross-sectional nature of the current data, we were unable to assess the causality of our findings, and longitudinal studies are needed to demonstrate the causal inference. Second, we used self-reported questionnaires to obtain information of SHS exposure, resulting in potential recall bias and exposure misclassification. This bias would likely to have been non-differential by the outcomes, and assuming the sensitivity plus the specificity for the exposure measurement was > 1, the results would have been biased towards the null. However, we observed mostly consistent dose-dependent associations across all SDSC domains, and the method we used remained to be the most cost-effective to assess SHS exposure in observational studies with large sample of children [32]. Third, measurement errors might occur for older children because the SDSC questionnaires were filled by their parents, who might be less likely to be aware of their sleeping status. However, the ‘accuracy-practicality’ trade-off exists in large population-based study, and it is more feasible for us to ensure identical and standard procedures across different primary and middle schools. Fourth, the prevalence of sleep problems and sleep-related symptoms in this study were smaller than the pooled prevalence reported by the results of the meta-analysis of Chinese studies [7]. However, our data was similar to those reported from the studies using the same measure of the SDSC [15, 33]. It should be noted that most previous studies used one question to extract information of sleep problems, and therefore the prevalence might be overestimated. Despite these limitations, our study had notable strengths, including a large sample size of Asian children with a wide range of age groups, comprehensive information on the exposure, outcomes and covariates, all which helped to improve the sufficient power of this study and strengthen the robustness of our findings. Most importantly, this is the first study to add evidence to examine the associations between early-life SHS exposure and various sleep outcomes in a vulnerable population of children.

## Conclusion

In conclusion, we found that children aged 6–18 years exposed to higher early-life SHS had higher odds of global sleep problems, and the associations were the strongest in children with symptom related to SWTD (i.e., sleep bruxism). Our findings highlight the importance to strengthen public health efforts and support the critical importance of maintaining a smoke-free environment especially in early life.

## Supplementary Information

**Additional file 1: eTable.1.** Basic information of previous observational studies regarding the associations of exposure to SHS with sleep-related problems in general children population^α^. **eTable.2.** Summaries of the results of previous observational studies regarding the associations of exposure to SHS with sleep-related problems in general children population. **eTable.3.** Distribution of the SDSC score in the participants. **eTable.4.** Associations of SHS exposure during early life using prenatal and early postnatal periods with the scores of the SDSC in children aged 6–18 years^a^. **eTable.5.** Associations of SHS exposure during early life using prenatal and early postnatal periods with the increased problems within the clinical range using the SDSC in children aged 6–18 years^a^. **eTable.6.** Associations of SHS exposure during early life with sleep problems in asthmatic children^a^. **eTable.7.** Associations of current SHS exposure with simultaneous sleep problems in children aged 6–18 years^a^.

## Data Availability

The datasets generated and/or analyzed during the current study are not publicly available but are available from the corresponding author on reasonable request.

## Abbreviations

SHS: Second-hand smoke
SDSC: Sleep Disturbance Scale for Children
DIMS: Disorders of initiating and maintaining sleep
SBD: Sleep breathing disorders
DA: Disorders of arousal
SWTD: Sleep-wake transition disorders
DOES: Disorders of excessive somnolence
SHY: Sleep hyperhidrosis
