# Supplementary material for: Exposure to second-hand smoke during early life and subsequent sleep problems in children: a population-based cross-sectional study
Source: Environ Health. 2021 Dec 18;20:127. doi: 10.1186/s12940-021-00793-0 (PMC8684187; doi:10.1186/s12940-021-00793-0)
Supplement: Supplementary file 1 — Additional file 1: eTable.1. Basic information of previous observational studies regarding the associations of exposure to SHS with sleep-related problems in general children populationα. eTable.2. Summaries of the results of previous observational studies regarding the associations of exposure to SHS with sleep-related problems in general children population. eTable.3. Distribution of the SDSC score in the participants. eTable.4. Associations of SHS exposure during early life using prenatal and early postnatal periods with the scores of the SDSC in children aged 6–18 yearsa. eTable.5. Associations of SHS exposure during early life using prenatal and early postnatal periods with the increased problems within the clinical range using the SDSC in children aged 6–18 yearsa. eTable.6. Associations of SHS exposure during early life with sleep problems in asthmatic childrena. eTable.7. Associations of current SHS exposure with simultaneous sleep problems in children aged 6–18 yearsa. [file 12940_2021_793_MOESM1_ESM.docx]

**Exposure to second-hand smoke during early life and subsequent sleep problems in children aged 6-18 years**

**eTable.1** Basic information of previous observational studies regarding the associations of exposure to SHS with sleep-related problems in general children population^α^

**eTable.2** Summaries of the results of previous observational studies regarding the associations of exposure to SHS with sleep-related problems in general children population

**eTable.3** Distribution of the SDSC score in the participants

**eTable.4** Associations of SHS exposure during early life using prenatal and early postnatal periods with the scores of the SDSC in children aged 6-18 years ^a^

**eTable.5** Associations of SHS exposure during early life using prenatal and early postnatal periods with the increased problems within the clinical range using the SDSC in children aged 6-18 years ^a^

**eTable.6** Associations of SHS exposure during early life with sleep problems in asthmatic children ^a^

**eTable.7** Associations of current SHS exposure with simultaneous sleep problems in children aged 6-18 years ^a^

This supplementary material has been provided by the authors to give readers additional information about their work.

**eTable.1** **Basic information of previous observational studies regarding the associations of exposure to SHS with sleep-related problems in general children population^α^**

| Author | Publish year | Study Period | Sample Size | Country | Study Design |
| --- | --- | --- | --- | --- | --- |
| Kim, et.al | 2020 | 2018.4 | 60,040 (13-18 yrs) | Korea | Cross-sectional |
| Lin, et.al | 2020 | 2009.6-2010-10 | 218 (10-15 yrs) | Taiwan, China | Cross-sectional |
| Włodarska, et.al | 2020 | NA | 70 Case & 90 Control: (6-18 yrs) | Poland | Case-control |
| Ramirez, et.al | 2020 | 1991.4-1992.12 | 12,030 (7 yrs) | the UK | Cohort |
| Khorasanchi, et.al | 2019 | 2015.1-20154 | 940 girls (12-17 yrs) | Iran | Cross-sectional |
| Morioka, et.al | 2018 | 2013.5 | 84,988 (13-18 yrs) | Japan | Cross-sectional |
| Chang, et.al | 2018 | 2009-2013 | 1,001 (14-17 yrs) | Canada | Cross-sectional |
| Reynaud, et.al | 2016 | 2003-2006 | 1,346 (5-6 yrs) | France | Cohort |
| Schwartz, et.al | 2014 | 2011 | 1,466 (13-18 yrs) | Canada | Cross-sectional |
| Kheirandish-Gozal, et.al | 2013 | 2007-2008 | 4,322 (6-12 yrs) | Iran | Cross-sectional |
| Zhu, et.al | 2013 | 2006 | 2.187 (2-6 yrs) | Hong Kong, China | Cross-sectional |
| Singh, et.al | 2013 | 2007.4-2008.7 | 63352 (6-17 yrs) | The USA | Cross-sectional |
| Gill, et.al | 2012 | 2008.3-2009.7 | 823 (3 yrs) | New Zealand | Cross-sectional |
| Beebe, et.al | 2012 | Since 2003.3 | 249 (2-3 yrs) | The USA | Cohort |
| Yılmaz, et.al | 2011 | 2008.5-2008.6 | 3441 (15-18 yrs) | Turkey | Cross-sectional |
| Li (a), et.al | 2010 | 2005.9-2005.12 | 21,052 (5-12 yrs) | China | Cross-sectional |
| Li (b), et.al | 2010 | NA | 6349 (5-14 yrs) | China | Cross-sectional |
| Sahin, et.al | 2009 | 2007.3-2007.6 | 1,164 (7-13 yrs) | Turkey | Cross-sectional |
| Gozal, et.al | 2008 | 1994-2004 | 16,321 (5-7 yrs) | The USA | Cross-sectional |
| Urschitz (a), et.al | 2004 | 2001.2-2001.12 | 1,144 (9.6±0.7 yrs) | Germany | Cross-sectional |
| Urschitz (b), et.al | 2007 | 2001 | 995 (7-12 yrs) | Germany | Cross-sectional |
| Kalra, et.al | 2006 | Since 2001 | 681 (1-2 yrs) | The USA | Cross-sectional |
| Kaditis, et.al | 2004 | NA | 3.666 (1-18 yrs) | Greece | Cross-sectional |
| Zhang, et.al | 2004 | 2002.3-2002.4 | 985 (4-12 yrs) | Australia | Cross-sectional |
| Castronovo, et.al | 2002 | 1998.10-1998.11 | 823 (3-6 yrs) | Italy | Cross-sectional |
| Brunetti, et.al | 2001 | NA | 895 (3-11 yrs) | Italy | Cross-sectional |
| Anuntaseree, et.al | 2001 | NA | 255 (6-13 yrs) | Thailand | Cross-sectional |

^α^ We only included studies with sample size >100.

**eTable.2** **Summaries of the results of previous observational studies regarding the associations of exposure to SHS with sleep-related problems in general children population**

| Specific type of SHS exposure and sleep domains | Study | Findings |
| --- | --- | --- |
| **Self-reported SHS, childhood, home** |  |  |
| DIMS, Insufficient sleep | Kim, et.al | **OR=1.42 (95%CI: 1.30-1.56)** |
| DIMS, insufficient sleep | Morioka, et.al | **OR=1.14 (95%CI: 1.08-1.20)** |
| DIMS, Inadequate sleep per week | Singh, et.al | **OR=1.23 (95%CI: 1.04-1.46)** |
| DIMS, difficulty initiating sleep | Morioka, et.al | **OR=1.08 (95%CI: 1.00-1.16)** |
| DIMS, difficulty maintaining sleep | Morioka, et.al | **OR=1.21 (95%CI: 1.11-1.31)** |
| DIMS, Insomnia | Morioka, et.al | **OR=1.16 (95%CI: 1.09-1.23)** |
| DIMS, Short sleep duration | Chang, et.al | **OR= 2.54 (95%CI: 1.25-5.16)** |
| DIMS, Inadequate sleep of weekdays | Singh, et.al | OR=1.07 (95%CI: 0.94-1.23) |
| DIMS, Sleep-onset insomnia of weekdays | Yılmaz, et.al | No associations |
| DIMS, Sleep-onset insomnia of weekends | Yılmaz, et.al | No associations |
| DIMS, short sleep duration | Morioka, et.al | OR=0.99 (95%CI: 0.93-1.05) |
| DIMS, Sleep duration of weekdays | Yılmaz, et.al | Negative (*P*=0.004) |
| DIMS, Sleep duration of weekends | Yılmaz, et.al | No associations |
| DA, early morning awaking | Morioka, et.al | OR=1.10 (95%CI: 0.98-1.23) |
| SBD, habitual snoring | Zhu, et.al | **OR=2.17 (95%CI: 1.01-4.70)** |
| Global problems, PSQI | Lin, et.al | OR=2.14 (95%CI: 0.75-6.12) |
| **Self-reported SHS, childhood, school** |  |  |
| DIMS, Insufficient sleep | Kim, et.al | **OR=1.26 (95%CI: 1.12-1.42)** |
| **Self-reported SHS, childhood, public areas** |  |  |
| DIMS, Insufficient sleep | Kim, et.al | **OR=1.87 (95%CI: 1.71-2.03)** |
| DIMS, difficulty initiating sleep | Morioka, et.al | **OR=1.10 (95%CI: 1.03-1.18)** |
| DIMS, Insomnia | Morioka, et.al | **OR=1.10 (95%CI: 1.04-1.16)** |
| DIMS, insufficient sleep | Morioka, et.al | **OR=1.20 (95%CI: 1.14-1.26)** |
| DIMS, short sleep duration | Morioka, et.al | **OR=1.11 (95%CI: 1.06-1.17)** |
| DIMS, difficulty maintaining sleep | Morioka, et.al | OR=1.06 (95%CI: 0.98-1.15) |
| DA, early morning awaking | Morioka, et.al | OR=0.98 (95%CI: 0.88-1.09) |
| **Self-reported SHS, childhood, areas not mentioned** |  |  |
| DIMS, Insomnia | Khorasanchi, et.al | **OR= 1.70 (95%CI: 1.20-2.30)** |
| DIMS, Short sleep duration | Schwartz, et.al | **OR= 2.51 (95%CI:1.59-3.98)** |
| DIMS, Restless sleep of weekdays | Schwartz, et.al | **β=-0.06 (95%CI: -0.01- -0.11)** |
| DIMS, Restless sleep of weekends | Schwartz, et.al | **β=-0.09 (95%CI: -0.03- -0.14)** |
| SBD, Sleep apnea | Khorasanchi, et.al | No group difference^α^ |
| DOES, Day time sleepiness | Khorasanchi, et.al | No group difference^α^ |
| **Parent-reported SHS, childhood, home** |  |  |
| DA, Waking up at night | Reynaud, et.al | **OR= 1.56 (95%CI: 1.00-2.48)** |
| DA, Waking up at night | Włodarska, et.al | No group difference^α^ |
| SBD, Sleep-Related Hypoxia | Urschitz (b), et.al | **OR=1.8 (95%CI: 1.1-2.8)** |
| SBD, Habitual Snoring | Urschitz (a), et.al | **OR=1.7 (95%CI: 1.1-2.6)** |
| SBD, Habitual Snoring | Gozal, et.al | **OR=1.39 (95%CI:1.25-1.54)** ^α^ |
| SBD, Habitual Snoring | Kheirandish-Gozal, et.al | **OR=2.25 (95%CI:1.44-3.66)** ^α^ |
| SBD, Habitual Snoring | Kaditis, et.al | **OR=1.4 (95%CI:1.1-1.8)** |
| SBD, Habitual Snoring | Kalra, et.al | OR=1.1 (95%CI: 0.8-2.0) |
| SBD, Habitual Snoring | Gill, et.al | OR=1.3 (95%CI: 0.8-2.4) |
| SBD, Higher AHI | Włodarska, et.al | **Group difference^α^** |
| SBD, Habitual Snorers | Li (b), et.al | OR=1.37 (95%CI: 0.77-2.44) |
| SBD, Habitual Snorers | Sahin, et.al | OR= 1.90 (95%CI: 0.99-3.65) |
| SBD, Habitual Snorers | Castronovo, et.al | No associations^α^ |
| SBD, Habitual Snorers | Li (a), et.al | No associations |
| SBD, Habitual Snorers | Brunetti, et.al | No group difference^α^ |
| SBD, Habitual Snoring | Anuntaseree, et.al | No associations |
| SBD, Habitual Snoring | Zhang, et.al | No group difference^α^ |
| SBD, Snoring | Włodarska, et.al | No group difference^α^ |
| SBD, Sleep apnea | Włodarska, et.al | No group difference^α^ |
| **Parent-reported SHS, childhood, areas not mentioned** |  |  |
| SBD, Mouth Breathing | Ramirez, et.al | **OR= 1.38 (95%CI:1.16-1.64)** |
| SBD, Snoring | Ramirez, et.al | **OR= 1.47 (95%CI:1.20-1.79)** |
| SBD, Apnea | Ramirez, et.al | **OR= 1.45 (95%CI:1.14-1.85)** |
| SBD, Any SBD symptoms | Ramirez, et.al | **OR= 1.46 (95%CI:1.26-1.70)** |
| **Parent-reported SHS, prenatal, areas not mentioned** |  |  |
| SBD, Mouth Breathing | Ramirez, et.al | **OR= 1.52 (95%CI:1.08-2.14)** |
| SBD, Snoring | Ramirez, et.al | **OR= 2.03 (95%CI:1.36-3.03)** |
| SBD, Apnea | Ramirez, et.al | **OR= 2.32 (95%CI:1.40-3.82)** |
| SBD, Any SBD symptoms | Ramirez, et.al | **OR= 1.73 (95%CI:1.26-2.37)** |
| **Urinary cotinine, childhood, areas not mentioned** |  |  |
| SBD, Habitual snoring | Zhu, et.al | **OR= 4.30 (95%CI:1.11-16.59)** |
| **Urinary cotinine, Prenatal, areas not mentioned** |  |  |
| SBD, Habitual snoring | Beebe, et.al | OR=1.00 (95%CI: 0.99-1.01) |

Abbreviations: DA, disorders of arousal; DIMS. disorders of initiating and maintaining sleep; DOES, disorders of excessive somnolence; PSQI, Pittsburg Sleep Quality Index; SBD, sleep breathing disorders; SHS, second-hand smoke.

Bold indicated significant results.

^α^ Results was obtained using crude model since there is no adjusted models in the relevant studies.

**eTable.3** **Distribution of the SDSC score in the participants**

|  | Raw score | | | T-score (mean±SD) | T-score>70  (Clinical range) | | |
| --- | --- | --- | --- | --- | --- | --- | --- |
|  | (mean±SD) | Min | Max |  | Boys | Girls | Total |
| Total score of the SDSC | 40.0±8.6 | 26 | 126 | 49.5±9.7 | 3.5% | 3.3% | 3.4% |
| DIMS* | 11.1±2.7 | 7 | 34 | 49.2±9.5 | 3.0% | 3.4% | 3.2% |
| SBD* | 3.9±1.4 | 3 | 15 | 49.6±9.6 | 6.6% | 4.2% | 5.4% |
| DA | 3.7±1.1 | 3 | 15 | 49.8±9.7 | 3.3% | 3.0% | 3.2% |
| SWTD | 10.5±3.1 | 6 | 30 | 49.6±9.9 | 4.4% | 4.7% | 4.5% |
| DOES* | 7.9±2.9 | 5 | 25 | 50.5±10.1 | 5.4% | 5.9% | 5.7% |
| SHY* | 2.9±1.3 | 2 | 10 | 49.2±9.4 | 6.9% | 3.7% | 5.3% |

Abbreviations: DA, disorders of arousal; DIMS. disorders of initiating and maintaining sleep; DOES, disorders of excessive somnolence; SBD, sleep breathing disorders; SD, standard deviation; SDSC, the Sleep Disturbance Scale for Children; SHS, second-hand smoke; SHY, sleep hyperhidrosis; SWTD, sleep–wake transition disorders.

*** Asterisk indicates statistically significant when comparing sex differences in the prevalence.**

**eTable.4** **Associations of SHS exposure during early life using prenatal and early postnatal periods with the scores of the SDSC in children aged 6-18 years ^a^**

| Estimate (95%CI) | Exposure to SHS during early life | | | | |
| --- | --- | --- | --- | --- | --- |
|  | During pregnancy | |  | From birth to 2 years | |
|  | Unexposed | Exposed |  | Unexposed | Exposed |
| Total score |  |  |  |  |  |
| Model 1 | [1] Reference | 3.83 (3.60, 4.05) |  | [1] Reference | 3.95 (3.73, 4.18) |
| Model 2 | [1] Reference | 3.60 (3.37, 3.83) |  | [1] Reference | 3.73 (3.51, 3.96) |
| DIMS score |  |  |  |  |  |
| Model 1 | [1] Reference | 2.46 (2.23, 2.68) |  | [1] Reference | 2.67 (2.45, 2.89) |
| Model 2 | [1] Reference | 2.24 (2.01, 2.47) |  | [1] Reference | 2.45 (2.23, 2.67) |
| SBD score |  |  |  |  |  |
| Model 1 | [1] Reference | 2.01 (1.79, 2.24) |  | [1] Reference | 1.93 (1.71, 2.16) |
| Model 2 | [1] Reference | 1.97 (1.74, 2.20) |  | [1] Reference | 1.90 (1.67, 2.12) |
| DA score |  |  |  |  |  |
| Model 1 | [1] Reference | 1.76 (1.53, 1.99) |  | [1] Reference | 2.02 (1.79, 2.24) |
| Model 2 | [1] Reference | 1.56 (1.32, 1.79) |  | [1] Reference | 1.82 (1.60, 2.05) |
| SWTD score |  |  |  |  |  |
| Model 1 | [1] Reference | 3.61 (3.38, 3.84) |  | [1] Reference | 3.66 (3.44, 3.89) |
| Model 2 | [1] Reference | 3.47 (3.23, 3.71) |  | [1] Reference | 3.53 (3.30, 3.76) |
| DOES score |  |  |  |  |  |
| Model 1 | [1] Reference | 2.85 (2.62, 3.09) |  | [1] Reference | 3.02 (2.79, 3.25) |
| Model 2 | [1] Reference | 2.65 (2.41, 2.89) |  | [1] Reference | 2.82 (2.59, 3.05) |
| SHY score |  |  |  |  |  |
| Model 1 | [1] Reference | 1.87 (1.65, 2.09) |  | [1] Reference | 1.64 (1.42, 1.85) |
| Model 2 | [1] Reference | 1.90 (1.68, 2.12) |  | [1] Reference | 1.67 (1.46, 1.89) |

Abbreviations: CI: confidence interval; DA, disorders of arousal; DIMS. disorders of initiating and maintaining sleep; DOES, disorders of excessive somnolence; SBD, sleep breathing disorders; SDSC, the Sleep Disturbance Scale for Children; SHS, second-hand smoke; SHY, sleep hyperhidrosis; SWTD, sleep–wake transition disorders.

^a^ Model 1 was adjusted for a school-level random intercept. Model 2 was adjusted for a school-level random intercept, child age, sex, only child, preterm birth and low birth weight, parental educational levels, yearly household income, maternal age during pregnancy, maternal smoking and alcohol consumption during pregnancy.

**eTable.5 Associations of SHS exposure during early life using prenatal and early postnatal periods with the increased problems within the clinical range using the SDSC in children aged 6-18 years ^a^**

| Odd ratios  (95%CI) | Exposure to SHS during early life | | | | |
| --- | --- | --- | --- | --- | --- |
|  | During pregnancy | |  | From birth to 2 years | |
|  | Unexposed | Exposed |  | Unexposed | Exposed |
| Increased sleep problems | |  |  |  |  |
| Model 1 | [1] Reference | 2.14 (1.92, 2.39) |  | [1] Reference | 2.17 (1.95, 2.42) |
| Model 2 | [1] Reference | 1.92 (1.71, 2.15) |  | [1] Reference | 1.94 (1.73, 2.16) |
| Increased DIMS |  |  |  |  |  |
| Model 1 | [1] Reference | 1.75 (1.56, 1.97) |  | [1] Reference | 1.85 (1.65, 2.07) |
| Model 2 | [1] Reference | 1.59 (1.41, 1.79) |  | [1] Reference | 1.68 (1.49, 1.89) |
| Increased SBD |  |  |  |  |  |
| Model 1 | [1] Reference | 1.69 (1.54, 1.85) |  | [1] Reference | 1.64 (1.49, 1.80) |
| Model 2 | [1] Reference | 1.65 (1.50, 1.82) |  | [1] Reference | 1.61 (1.46, 1.77) |
| Increased DA |  |  |  |  |  |
| Model 1 | [1] Reference | 1.54 (1.36, 1.73) |  | [1] Reference | 1.63 (1.45, 1.83) |
| Model 2 | [1] Reference | 1.38 (1.22, 1.56) |  | [1] Reference | 1.46 (1.30, 1.65) |
| Increased SWTD |  |  |  |  |  |
| Model 1 | [1] Reference | 1.90 (1.72, 2.09) |  | [1] Reference | 1.91 (1.74, 2.11) |
| Model 2 | [1] Reference | 1.77 (1.60, 1.96) |  | [1] Reference | 1.79 (1.62, 1.97) |
| Increased DOES |  |  |  |  |  |
| Model 1 | [1] Reference | 1.80 (1.65, 1.97) |  | [1] Reference | 1.81 (1.66, 1.98) |
| Model 2 | [1] Reference | 1.69 (1.54, 1.85) |  | [1] Reference | 1.69 (1.55, 1.86) |
| Increased SHY |  |  |  |  |  |
| Model 1 | [1] Reference | 1.68 (1.53, 1.85) |  | [1] Reference | 1.51 (1.37, 1.66) |
| Model 2 | [1] Reference | 1.71 (1.55, 1.88) |  | [1] Reference | 1.53 (1.39, 1.69) |
| Shorter sleep duration | |  |  |  |  |
| Model 1 | [1] Reference | 1.02 (0.93, 1.12) |  | [1] Reference | 1.11 (1.02, 1.22) |
| Model 2 | [1] Reference | 1.01 (0.91, 1.12) |  | [1] Reference | 1.09 (0.99, 1.20) |
| Longer sleep latency | |  |  |  |  |
| Model 1 | [1] Reference | 1.63 (1.35, 1.97) |  | [1] Reference | 1.67 (1.38, 2.01) |
| Model 2 | [1] Reference | 1.47 (1.21, 1.79) |  | [1] Reference | 1.49 (1.23, 1.80) |

Abbreviations: CI: confidence interval; DA, disorders of arousal; DIMS. disorders of initiating and maintaining sleep; DOES, disorders of excessive somnolence; SBD, sleep breathing disorders; SDSC, the Sleep Disturbance Scale for Children; SHS, second-hand smoke; SHY, sleep hyperhidrosis; SWTD, sleep–wake transition disorders.

^a^ Model 1 was adjusted for a school-level random intercept. Model 2 was adjusted for a school-level random intercept, child age, sex, only child, preterm birth and low birth weight, parental educational levels, yearly household income, maternal age during pregnancy, maternal smoking and alcohol consumption during pregnancy.

**eTable.6 Associations of SHS exposure during early life with sleep problems in asthmatic children ^a^**

|  | Exposure to SHS during the first 1000 days of life | | |
| --- | --- | --- | --- |
|  | Unexposed | Ever exposed | Always exposed |
| Continuous outcomes [Estimate (95%CI)] |  |  |  |
| Total score | [1] Reference | 2.60 (2.30, 2.90) | 4.30 (4.03, 4.57) |
| DIMS score | [1] Reference | 1.51 (1.21, 1.80) | 2.82 (2.56, 3.08) |
| SBD score | [1] Reference | 1.21 (0.91, 1.50) | 2.16 (1.90, 2.43) |
| DA score | [1] Reference | 1.33 (1.03, 1.63) | 1.97 (1.70, 2.24) |
| SWTD score | [1] Reference | 2.60 (2.30, 2.91) | 4.06 (3.78, 4.33) |
| DOES score | [1] Reference | 2.03 (1.72, 2.33) | 3.26 (2.98, 3.53) |
| SHY score | [1] Reference | 1.19 (0.91, 1.47) | 2.04 (1.79, 2.30) |
| Binary outcomes [Odd Ratios (95%CI)] |  |  |  |
| Increased sleep problems | [1] Reference | 1.66 (1.42, 1.94) | 2.09 (1.83, 2.38) |
| Increased DIMS | [1] Reference | 1.43 (1.21, 1.69) | 1.78 (1.55, 2.04) |
| Increased SBD | [1] Reference | 1.45 (1.27, 1.66) | 1.77 (1.58, 1.99) |
| Increased DA | [1] Reference | 1.45 (1.23, 1.70) | 1.49 (1.29, 1.72) |
| Increased SWTD | [1] Reference | 1.45 (1.26, 1.67) | 1.97 (1.75, 2.21) |
| Increased DOES | [1] Reference | 1.45 (1.28, 1.65) | 1.84 (1.65, 2.05) |
| Increased SHY | [1] Reference | 1.40 (1.23, 1.61) | 1.75 (1.56, 1.97) |
| Shorter sleep duration | [1] Reference | 1.06 (0.93, 1.21) | 1.05 (0.94, 1.18) |
| Longer sleep latency | [1] Reference | 1.50 (1.16, 1.93) | 1.51 (1.20, 1.90) |

Abbreviations: CI: confidence interval; DA, disorders of arousal; DIMS. disorders of initiating and maintaining sleep; DOES, disorders of excessive somnolence; SBD, sleep breathing disorders; SDSC, the Sleep Disturbance Scale for Children; SHS, second-hand smoke; SHY, sleep hyperhidrosis; SWTD, sleep–wake transition disorders.

^a^ Model was adjusted for a school-level random intercept, child age, sex, only child, preterm birth and low birth weight, parental educational levels, yearly household income, maternal age during pregnancy, maternal smoking and alcohol consumption during pregnancy.

**eTable.7** **Associations of current SHS exposure with simultaneous sleep problems in children aged 6-18 years ^a^**

|  | current SHS Exposure | |
| --- | --- | --- |
|  | Unexposed | Exposed |
| Continuous outcomes [Estimate (95%CI)] |  |  |
| Total score | [1] Reference | 2.08 (1.89, 2.26) |
| DIMS score | [1] Reference | 1.31 (1.13, 1.49) |
| SBD score | [1] Reference | 1.10 (0.91, 1.28) |
| DA score | [1] Reference | 0.88 (0.69, 1.06) |
| SWTD score | [1] Reference | 2.07 (1.88, 2.25) |
| DOES score | [1] Reference | 1.54 (1.35, 1.73) |
| SHY score | [1] Reference | 0.94 (0.77, 1.12) |
| Binary outcomes [Odd Ratios (95%CI)] |  |  |
| Increased sleep disturbance | [1] Reference | 1.42 (1.28, 1.58) |
| Increased DIMS | [1] Reference | 1.37 (1.23, 1.52) |
| Increased SBD | [1] Reference | 1.27 (1.17, 1.38) |
| Increased DA | [1] Reference | 1.10 (0.99, 1.23) |
| Increased SWTD | [1] Reference | 1.36 (1.24, 1.49) |
| Increased DOES | [1] Reference | 1.34 (1.23, 1.45) |
| Increased SHY | [1] Reference | 1.29 (1.19, 1.41) |
| Shorter sleep duration | [1] Reference | 1.01 (0.93, 1.09) |
| Longer sleep latency | [1] Reference | 1.40 (1.18, 1.66) |

Abbreviations: CI: confidence interval; DA, disorders of arousal; DIMS. disorders of initiating and maintaining sleep; DOES, disorders of excessive somnolence; SBD, sleep breathing disorders; SDSC, the Sleep Disturbance Scale for Children; SHS, second-hand smoke; SHY, sleep hyperhidrosis; SWTD, sleep–wake transition disorders.

^a^ Model was adjusted for a school-level random intercept, child age, sex, only child, preterm birth and low birth weight, parental educational levels, yearly household income, maternal age during pregnancy, maternal smoking and alcohol consumption during pregnancy.
